# A Genome-Wide Scan for Breast Cancer Risk Haplotypes among African American Women

**DOI:** 10.1371/journal.pone.0057298

**Published:** 2013-02-28

**Authors:** Chi Song, Gary K. Chen, Robert C. Millikan, Christine B. Ambrosone, Esther M. John, Leslie Bernstein, Wei Zheng, Jennifer J. Hu, Regina G. Ziegler, Sarah Nyante, Elisa V. Bandera, Sue A. Ingles, Michael F. Press, Sandra L. Deming, Jorge L. Rodriguez-Gil, Stephen J. Chanock, Peggy Wan, Xin Sheng, Loreall C. Pooler, David J. Van Den Berg, Loic Le Marchand, Laurence N. Kolonel, Brian E. Henderson, Chris A. Haiman, Daniel O. Stram

**Affiliations:** 1 Department of Preventive Medicine, Keck School of Medicine and Norris Comprehensive Cancer Center, University of Southern California, Los Angeles, California, United States of America; 2 Department of Epidemiology, Gillings School of Global Public Health, and Lineberger Comprehensive Cancer Center, University of North Carolina, Chapel Hill, North Carolina, United States of America; 3 Department of Cancer Prevention and Control, Roswell Park Cancer Institute, Buffalo, New York, United States of America; 4 Cancer Prevention Institute of California, Fremont, California, United States of America; 5 Stanford University School of Medicine and Stanford Cancer Institute, Stanford, California, United States of America; 6 Division of Cancer Etiology, Department of Population Science, Beckman Research Institute, City of Hope, Duarte, California, United States of America; 7 Division of Epidemiology, Department of Medicine, Vanderbilt Epidemiology Center, and Vanderbilt-Ingram Cancer Center, Vanderbilt University School of Medicine, Nashville, Tennessee, United States of America; 8 Sylvester Comprehensive Cancer Center and Department of Epidemiology and Public Health, University of Miami Miller School of Medicine, Miami, Florida, United States of America; 9 Epidemiology and Biostatistics Program, Division of Cancer Epidemiology and Genetics, National Cancer Institute, Bethesda, Maryland, United States of America; 10 The Cancer Institute of New Jersey, New Brunswick, New Jersey, United States of America; 11 Department of Pathology, Keck School of Medicine and Norris Comprehensive Cancer Center, University of Southern California, Los Angeles, California, United States of America; 12 Epigenome Center, Norris Comprehensive Cancer Center, University of Southern California, Los Angeles, California, United States of America; 13 Epidemiology Program, University of Hawaii Cancer Center, Honolulu, Hawaii, United States of America; University of Illinois at Chicago, United States of America

## Abstract

Genome-wide association studies (GWAS) simultaneously investigating hundreds of thousands of single nucleotide polymorphisms (SNP) have become a powerful tool in the investigation of new disease susceptibility loci. Haplotypes are sometimes thought to be superior to SNPs and are promising in genetic association analyses. The application of genome-wide haplotype analysis, however, is hindered by the complexity of haplotypes themselves and sophistication in computation. We systematically analyzed the haplotype effects for breast cancer risk among 5,761 African American women (3,016 cases and 2,745 controls) using a sliding window approach on the genome-wide scale. Three regions on chromosomes 1, 4 and 18 exhibited moderate haplotype effects. Furthermore, among 21 breast cancer susceptibility loci previously established in European populations, 10p15 and 14q24 are likely to harbor novel haplotype effects. We also proposed a heuristic of determining the significance level and the effective number of independent tests by the permutation analysis on chromosome 22 data. It suggests that the effective number was approximately half of the total (7,794 out of 15,645), thus the half number could serve as a quick reference to evaluating genome-wide significance if a similar sliding window approach of haplotype analysis is adopted in similar populations using similar genotype density.

## Introduction

Genome-wide association studies (GWAS) have been demonstrated to have the power to detect modest to small effects of genetic variants with various common diseases [1]. A large number of novel SNPs have been identified and successfully replicated in associations with complex diseases, such as cancers, diabetes, and cardiovascular disease [2]. Meanwhile, haplotype analysis has become a prominent example of multilocus genetic association studies and has assisted in finding new disease susceptibility loci [3]–[8]. Haplotypes consist of SNPs or other genetic markers on the same chromosome that are inherited together with little contemporary recombination [9]. Haplotype information may aid GWAS in identifying new marker-phenotype associations for several reasons [10]. First, haplotypes characterize the exact organization of alleles along the chromosome. Although D’ and r^2^ are useful in capturing the linkage disequilibrium (LD) pattern between a pair of markers, they are hardly to be extended to higher order of dependency among markers. As a result, LD analysis based on underlying haplotypes can be more accurate [11]. Second, by constructing haplotype blocks from SNPs, more information can be incorporated into the association tests, especially when haplotypes themselves are in closer LD with the causal variant than any single genotyped SNP [12]. Haplotype analysis has been reported to be superior to analysis based on individual SNPs by simulation [13] and empirical studies [14], [15].

Although haplotype analysis is seemingly appealing, its implementation on the genome wide scale is unwieldy given the uncertainty and complexity of haplotypes [16], as well as the difficulty of adjusting for multiple testing when hundreds of thousands of hypotheses are being tested simultaneously. For instance, there is no consensus in the exact definition of haplotype blocks, making the boundaries of haplotype blocks not unambiguous [17]. One definition is based on D’ among neighboring SNPs which needs to exceed a pre-specified cutoff value [18]; another commonly implemented method requires a reduced haplotype diversity on a chromosomal segment [19]. Unfortunately, no method is uniformly better than the others in application [15]. We favor a sliding window framework since haplotypes can be quickly constructed and all genotyped SNPs are incorporated [20]. Fixed window sizes are computationally easier and more efficient in practice relative to varying window sizes. Mathias et al [21] successfully identified five asthma susceptibility loci on chromosome 11 in African Americans via the sliding window approach, in which the window sizes were 2–6 SNPs. Lambert et al. [22] adopted a similar approach where 10 consecutive haplotype tagging SNPs (htSNPs) were defined as a sliding window and found a haplotype residing in *FRMD4A* gene at 10p13 with increased risk for Alzheimer’s disease. In this paper, we scanned throughout the 22 autosomes to search for significant haplotype effects for breast cancer risk among 5,761 African American women using the sliding window approach of 5 contiguous SNPs. The haplotype effects were then compared with individual SNP effects including genotyped and imputed SNPs at the same chromosomal position. To determine a valid significance level, 1,000 permutations were exploited using the chromosome 22 data. The permutation-based chromosome-wide significance level for chromosome 22 and the effective number of independent tests were computed from the empirical distribution of the minimum p-values. The genome-wide significance level can then be readily determined through Bonferroni correction by substituting the effective number of tests for the total number of tests. While globally significant results were not obtained, closer attention should be paid to the regions revealed by the most significant haplotypes on chromosomes 1, 4 and 18. We also scrutinized 21 known breast cancer risk regions [23] for potential haplotype effects and found 10p15 and 14q24 may possess novel haplotype effects.

## Materials and Methods

### Ethics Statement

The Institutional Review Board at the University of Southern California approved the study protocol. All participants gave informed written consent at the time of blood draw.

### Study Population

There were a total of 5,984 African American women included in this study, of which 3,153 were cases with breast cancer and 2,831 were controls. The entire sample was derived from nine epidemiological studies: (i) The Multiethnic Cohort Study (MEC) [24]: 734 cases and 1,003 controls; (ii) The Los Angeles component of the Women’s Contraceptive and Reproductive Experiences Study (CARE) [25]: 380 cases and 224 controls; (iii) The Women’s Circle of Health Study (WCHS) [26]: 272 cases and 240 controls; (iv) The San Francisco Bay Area Breast Cancer Study (SFBCS) [27]: 172 cases and 231 controls; (v) The Northern California Breast Cancer Family Registry (NC-BCFR) [28]: 440 cases and 53 controls; (vi) The Carolina Breast Cancer Study (CBCS) [29]: 656 cases and 608 controls; (vii) The Prostate, Lung, Colorectal, and Ovarian Cancer Screening Trial (PLCO) Cohort [30]: 64 cases and 133 controls; (viii) The Nashville Breast Health Study (NBHS) [31]: 310 cases and 186 controls; (ix) Wake Forest University Breast Cancer Study (WFBC) [32]: 125 cases and 153 controls. All cases were African American women diagnosed with invasive or in situ breast cancer. Controls were mainly recruited through random digit dialing. A more detailed description of the characteristics of each study is available in Table S1 and elsewhere [23].

### Genotyping and Quality Control

Genotyping was performed using the Illumina Human 1M-Duo chip. Individuals whose samples had low DNA concentrations (<20 ng/µl) were removed (n = 52). We also removed unexpectedly related individuals (n = 29), call rates <95% (n = 100), African ancestry <5% (n = 36), and individuals of ambiguous sex (n = 6). We excluded SNPs with call rate <95% (n = 21,732) and minor allele frequency (MAF) <1% (n = 80,193). SNPs with a concordance rate lower than 98% were removed too (n = 11,701). The average concordance rate of the sample was 99.95%. Hardy-Weinberg equilibrium (HWE) was not imposed as one of the quality control criteria given that African Americans are known as an admixed population [33]. Except for a SNP on chromosome 5 showing significant deviation from HWE (discussion follows in the Results section), none of the other SNPs included in the following analyses were severely out of HWE (Exact test p-value >1×10^−6^) [34]. The total number of SNPs remained in the analysis was 1,006,480 in 5,761 subjects (3,016 cases and 2,745 controls).

### Statistical Analysis

#### Sliding window size

The sliding window approach was adopted to define haplotype blocks throughout 22 autosomes for its maximum coverage of genotyped SNPs given the exploratory nature of the present study. The choice of the 5-SNP window was mostly in agreement with the average block size for the HapMap Yoruba population [in Ibadan, Nigeria (YRI), HapMap Phase II] (Table S2). Wang et al [35] showed that based on Gabriel’s definition of haplotype blocks [9], [36], 57% of LD blocks in the YRI population were shorter than 10 kb and 37% of the blocks were between 10 kb and 50 kb. For our AABC data, the universal 5-SNP windows across 22 chromosomes achieved a comparable distribution of haplotype block sizes, i.e., 55% of the 5-SNP windows shorter than 10 kb and 44% between 10 kb and 50 kb long. The distributions of haplotype block sizes defined by sliding windows did not differ greatly by chromosome (Figure S1), indicating that on no chromosome the 5-SNP sliding windows have a disproportionately poor coverage of exceptionally long or short blocks in general. Admittedly, the universal 5-SNP windows across 22 autosomes or throughout approximately 1 million SNPs may not comprehensively capture individual haplotype block size variations at specific loci. It is nonetheless deemed a fairly good approximation with some theoretical basis.

#### Haplotype inference

The haplotype frequencies within each haplotype block defined by the sliding windows were estimated using the Expectation-Maximization (E-M) algorithm outlined by Excoffier and Slatkin [37], and Stram [38], [39]. Let 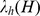 count the true, yet generally unknown, number of copies of a haplotype *h*, with frequency 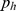, contained in the haplotype pair *H* carried by a given individual, i.e., 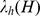 takes possible values of 0, 1 or 2, meaning 0, 1 or 2 copies of such haplotype *h* in haplotype pair *H* are inherited from parents; let 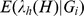 denote the expected number of copies of each possible haplotype *h* given the individual’s observed genotype 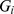. These expectations are computed iteratively as
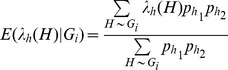
(1)with
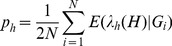
(2)where 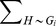 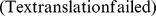
indicates the summation is over the haplotype pairs, 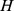, compatible with the observed genotype,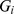. The algorithm starts with initial haplotype frequencies, 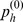, and updates them iteratively. Equation (1) is the expectation step and (2) is the maximization step of the E-M algorithm.

#### Association testing

The inferred haplotype dosage estimates, 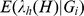, abbreviated as 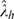, can be used individually in a 1-degree-of-freedom (d.f.) test in testing for haplotype-specific associations with the disease using model (3),
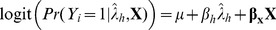
(3)or a global test simultaneously fitting all haplotypes 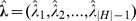 within the haplotype block defined by a sliding window using model (4),
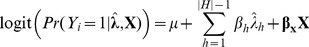
(4)where 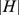 denotes the total number of possible haplotypes within that block and the degrees of freedom of the global test in model (4) are therefore 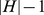. In both models, X is the vector of covariates, including age, study, and the top ten eigenvectors of ancestral information estimated by principal components analysis [40] to adjust for global ancestry differences. The eigenvectors are included in the model to control for potential confounding due to population stratification and admixture. In haplotype association analysis, a large fraction of the inferred haplotypes can be very rare, with frequency close to zero [41]. It is customary to discard rare haplotypes that are less than 1% frequent to reduce the total d.f. of the model so that the power to detect risk effects of relatively common haplotypes can be well preserved. Suppose that there are 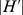 haplotypes greater than 1% of frequency, where 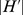
<<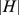 holds true in many cases, the d.f. of the global test reduces to 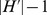 from 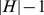 as indicated in model (5)



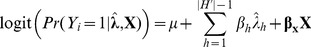
(5)We started with applying the global test throughout the whole genome to agnostically search for haplotype effects following the 5-SNP sliding window framework, while the 1 d.f. test of individual haplotype-specific effects was performed only when a potentially significant region was detected by the global test. For visualization purposes, haplotype effects were compared to the effects of the constituent SNPs at the same chromosomal region by an overlaid Manhattan plot showing the statistical significance, presented as –log_10_(p-value), of both haplotypes and SNPs. Haplotype effects would become interesting only if a noticeable haplotype effect peak was not accompanied by a similar significance peak involving the constituent SNPs. For regions exhibiting considerable haplotype effects, they were further extended both upstream and downstream by half of the original width to include more flanking SNPs and haplotypes, making the extended regions twice longer (Table S3). All possible individual haplotypes composed of 2 up to 10 SNPs (or the maximum number of genotyped SNPs contained in the extended region, whichever is smaller) with haplotype frequency >1% were investigated exhaustively to single out the particular haplotype(s) explaining the significant global test. The top individual haplotypes were further verified by a likelihood ratio (LR) test comparing the model with both the top haplotype and the best single SNP contained (model 6) to the nested model with the same best SNP only (model 7),
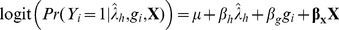
(6)

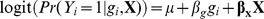
(7)where *g_i_* denotes the genotypes of the SNP carried by an individual *i* and an additive excessive effect of each risk allele on the disease is assumed. The novelty of the haplotype effects compared to the SNP effects was assessed using a LR test with 1 d.f. We were also interested in whether the haplotype effects could be otherwise captured by genotype imputation in the same region. The genotype imputation was performed by Mendel-GPU [42] using the 1000 Genomes Projects (1 KGP) data as the reference panel [43]. The much denser 1 KGP has a better genomic coverage of rare and low frequency markers and is reported to be capable of providing more statistical power to identify the underlying associations [44]. The superiority of haplotype analysis to SNP imputation could be highlighted by the presence of haplotype signals where significant genotyped or imputed SNPs are absent. In regions with the strongest haplotype effects, we also inferred and adjusted for the local ancestry information for each marker residing near the haplotypes of interest (±250 kb). The local ancestry characterizes the proportions of European and African ancestry, represented by the posterior probabilities of carrying 0, 1, and 2 copies of a European allele at each SNP. The local ancestry was computed by HAPMIX [45] with 240 HapMap EUR+YRI phased founder haplotypes per chromosome as input. The top haplotype effect was further adjusted for the inferred local ancestry in addition to adjustment for global ancestry (i.e. using the leading principal components), age, and study as described above. This additional adjustment for local ancestry could help eliminate false positive haplotype effects that were confounded by local ancestry [46].

In addition, haplotype effects in the neighborhood of known breast cancer risk SNPs identified predominantly in European populations were investigated especially carefully. Twenty-one regions (1p11, 2q35, 3p24, 5p12, 5q11, 6q14, 6q25, 8q24, 9p21, 9q31, 10p15, 10q21, 10q22, 10q26, 11p15, 11q13, 14q24, 16q12, 17q22, 19p13, and 20q11) and their associated SNPs were of primary interest. Regions with potential of harboring unknown haplotype effects were scrutinized by inferring all possible individual haplotypes of frequency >1% consisting of 2–10 consecutive SNPs in the neighborhood of ±250 kb of known breast cancer risk hits (except for 8q24, where ±2 Mb was used [47]–[49]). As before, the important haplotype effects were compared with the significance of genotyped as well as with the 1 KGP imputed SNPs in the same region. The independence of these haplotype-disease associations were further verified by LR tests adjusting for the SNP effects from both the regionally best SNP and the known breast cancer risk SNP. Notable haplotypes residing in proximity to the known breast cancer risk hits were again corrected for local ancestry inferred from the same region to eliminate potential confounding due to local genetic ancestry admixture.

PLINK [50] was the primary software to conduct the association analyses. All regression models were adjusted for age, study, and global ancestry. For important haplotypes indentified through association analyses, local ancestry was additionally adjusted for.

#### Permutation test

In order to obtain a valid significance threshold for the global test of haplotype analysis, 1,000 replicates of chromosome 22 data were generated by randomly shuffling the case-control status for each individual in the sample while maintaining the same numbers of cases and controls as in the original data. Each replicate was analyzed using the same global test logistic regression model to test the overall significance of haplotype blocks defined by the same 5-SNP sliding window (model 5). The same covariates were adjusted for as well, i.e., age, study and global ancestry, but not local ancestry. The minimum p-values of the global tests for haplotype block effects from 1,000 permutations were recorded and sorted in ascending order and the fifth percentile of the 1,000 minimum p-values was considered the permutation-based p-value so that the chromosome-wide type I error rate equals 0.05. Following Dudbridge et al. [51], we substituted the total number of tests with the effective number of independent tests n_eff_. If n_eff_ exists, then it can be inferred from the beta distribution of the minimum p-values with parameters (1,n_eff_ ) [52].
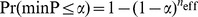



The probability density function of the beta distribution with parameters 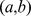 is,
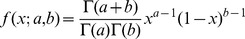
where 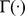 is the gamma function with two parameters 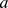,
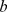
>0. Therefore beta distributions were fitted to the minimum p-values from the 1,000 permutation replicates in two scenarios: (i) the parameter 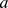 of the beta distribution is set equal to 1; (ii) both parameters 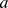 and 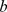 are free to vary. In the second scenario, the minimum p-values are consistent with the theoretical beta distribution if the null hypothesis 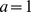 is not rejected; 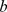 can thus be interpreted as the effective number of independent sliding windows, n_eff_. The parameters in the beta distribution were estimated using maximum likelihood estimation (MLE) method. Quantile-Quantile (QQ) plots were generated to evaluate the goodness-of-fit of these beta distributions. The aforementioned analysis was implemented in SAS version 9.1.2 (SAS Institute, Cary, NC).

## Results

**Figure 1:**
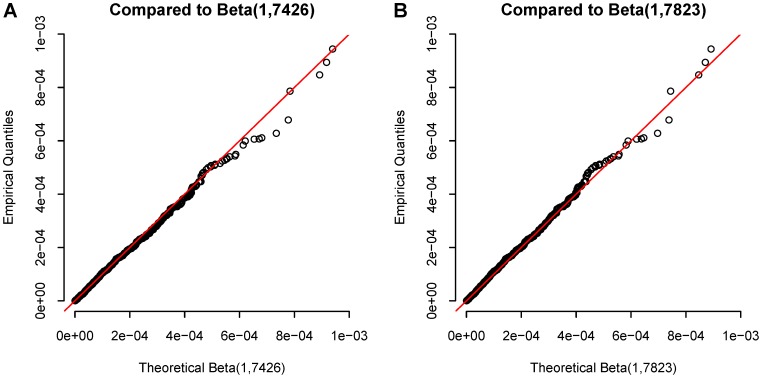
Comparison of the permutation minimum p-values to theoretical beta distributions. ( A). Quantile-Quantile plot comparing the minimum p-values from 1,000 permutations on chromosome 22 data to beta(1,7426). (B). Quantile-Quantile plot comparing the minimum p-values to beta(1,7823).

The minimum p-values from the 1,000 permutations of chromosome 22 data containing 15,649 genotyped SNPs ranged between 1.54×10^−7^ and 9.44×10^−4^ with the fifth percentile being 5.58×10^−6^. So the permutation-based effective number of tests for chromosome 22 was simply 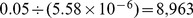. The maximum likelihood estimates of the beta distribution parameters were 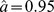 and 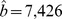; or 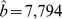 if 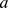 was constrained at 1. Although the null hypothesis of equality 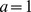 was nominally rejected in the former two-parameter case (p<0.01), 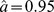 was close to 1 and the QQ plot comparing it to the Beta(1,7426) distribution showed the majority of the data points fell on the diagonal line, suggesting the lack of fit was not severe (Figure 1A). When setting 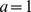 and experimenting with different
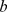
’s, i.e.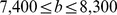, goodness-of-fit tests based on empirical distribution functions (EDF) statistics (Kolmogorov-Smirnov, Cramer-von Mises and Anderson-Darling statistics) did not reject the null hypothesis at the 0.10 significance level, implying that the minimum p-values followed the designated beta distributions satisfactorily (Table 1). The range of the effective numbers of tests, 7,400–8,300, included half the number of total sliding windows (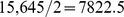). The corresponding significance level under this approximation was 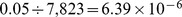, benchmarking to the 5.7 percentile of the minimum p-values from 1,000 permutations. The QQ plot for those minimum p-values compared to Beta (1,7823) distribution indicated the fit was reasonably good (Figure 1B) and none of the goodness-of-fit tests were rejected (p>0.25). We proceeded with the effective number of independent tests equal to half of the total number of overlapping haplotype blocks as a quick reference to spotting potentially significant haplotype effects. The genome-wide significance level was therefore derived as 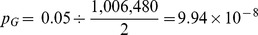
in contrast to the Bonferroni corrected genome-wide significance level 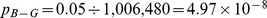.

**Table 1:** Fitting the minimum p-values from 1,000 permutations of chromosome 22 data to theoretical beta distributions beta(a,b).

Parameter		Goodness of fit (p-value)		
a	b	Kolmogorov-Smirnov	Cramér-von Mises	Anderson-Darling
0.95	7426	>0.25	>0.25	>0.25
1	7794	>0.25	>0.25	>0.25
1	8500	0.044	0.061	0.039
1	8400	0.088	0.123	0.089
1	8300	0.164	0.236	0.188
1	7600	>0.25	>0.25	>0.25
1	7400	>0.25	0.16	0.116
1	7300	0.147	0.049	0.047

**Figure 2:**
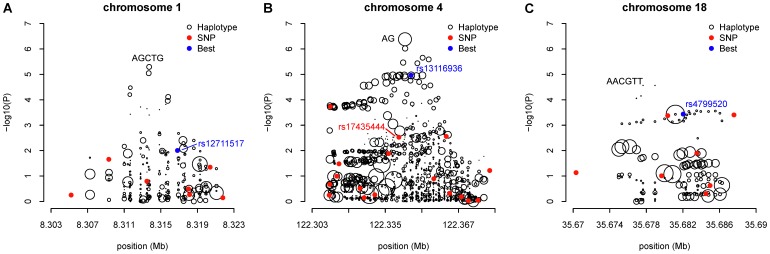
Comparison of the significance of individual haplotypes with the most significant SNPs in three regions on chromosomes 1, 4 and 18. These three regions, namely, (A) chr1∶8,309,317-8,318,147; (B) chr4∶122,325,743- 122,363,114; and (C) chr18∶35,670,316-35,683,522 were identified by the genome-wide haplotype association analysis using 5-SNP sliding windows. The regions were further extended both upstream and downstream by half of the original width to explore underdetected effects. Black circles denote individual haplotypes, the sizes of which are proportional to their haplotype frequencies. Red dots denote genotyped SNPs within the same region. Blue dot shows the most significant SNP.

**Figure 3:**
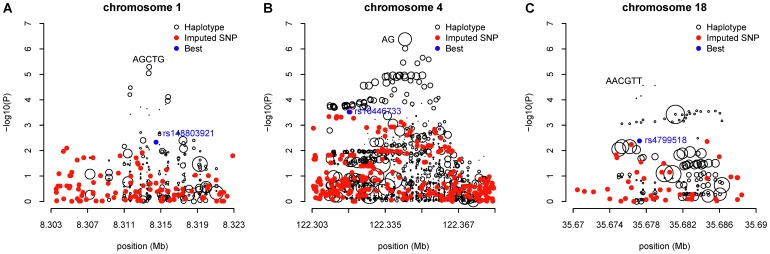
Comparison of the significance of individual haplotypes with imputed SNPs in regions on chromosomes 1, 4 and 18. Contrast of the haplotype effects with the effects of the 1000 Genome Project imputed SNPs in these three regions, namely, (A) chr1∶8,309,317-8,318,147; (B) chr4∶122,325,743- 122,363,114; and (C) chr18∶35,670,316-35,683,522 are shown. Black circles denote individual haplotypes, the sizes of which are proportional to their haplotype frequencies. Red dots denote imputed SNPs within the same region. Blue dot shows the most significant imputed SNP.

In search of haplotype peaks where significant SNPs were absent on the Manhattan plots, a region on chromosome 5 exhibited a distinct haplotype effect compared with individual SNP associations at the same chromosomal region (Figure S2). There were five overlapping haplotype blocks defined by 5-SNP sliding windows with global test p-values (p = 1.70×10^−8^, 3.16×10^−8^, 1.85×10^−7^, 1.45×10^−6^, and 3.38×10^−6^, respectively) less than any single SNP’s p-value within the same region. However, the most significant SNP rs6882564 (p = 1.14×10^−4^) made up all the significant haplotypes and were noted to be severely out of HWE (p<1×10^−7^). A review of the intensity plots for this SNP showed that rs6882564 was clearly miscalled by the genotyping algorithm, and thus we dropped from consideration all haplotypes that contain rs6882564, leaving no other haplotypes in the same region genome-wide significant. No other haplotype blocks throughout the genome had a global test p-value less than 10^−6^. The top 10 independent genomic regions with haplotype global test p-value between 1.60×10^−6^ and 1.51×10^−5^ are summarized in Table S3. After visual examination of the Manhattan plots contrasting the haplotype-specific effects with the individual SNP effects, the remaining most significant regions unlikely to be explained solely by SNPs were chr1∶8,309,317-8,318,147, chr4∶122,325,743-122,363,114, and chr18∶35,670,316-35,683,522. Notably, on chromosome 1, the 5-SNP haplotype AGCTG (Position: 8309317-8318147; frequency = 0.24) (Figure 2A; Table 2) comprised of SNPs rs9628987, rs2289731, rs12711517, rs2305016, and rs7535752, had a p-value three orders of magnitude less than that of the most significant SNP contained in the haplotype, rs12711517 (haplotype p = 5.09×10^−6^ vs. SNP p = 9.88×10^−3^). When conditioning on this locally most significant SNP, the haplotype effect stayed almost unchanged (adjusted OR = 0.82; 95% CI = 0.74–0.91) and remained the most significant haplotype, although the adjusted haplotype specific association p-value was less significant than that of without adjustment for the best SNP (unadjusted haplotype p = 5.09×10^−6^ vs. adjusted haplotype p = 1.36×10^−4^). On chromosome 4, a 2-SNP haplotype AG (Position: 122340944-122346258; frequency = 0.64) was close to two orders of magnitude more significant than its best individual SNP, rs13116936 (3.37×10^−7^ vs. 1.09×10^−5^) (Figure 2B) and the unadjusted haplotype specific effect was among the most significant in all top 10 independent regions. After adjusting for the best SNP, the haplotype effect remained significant at p = 7.54×10^−4^. A potentially interesting finding was on chromosome 18 (Figure 2C) where a much rarer 6-SNP haplotype AACGTT (Position: 35670316-35684521; frequency = 0.03) showed an improvement of haplotype significance with the adjusted p-value of 2.42×10^−5^ in contrast to the unadjusted p-value of 6.96×10^−5^. The haplotype specific effect did not alter meaningfully before and after the adjustment for the best SNP (unadjusted OR = 1.72, 95% CI = 1.32-2.25; adjusted OR = 1.79, 95% CI = 1.36-2.34). The carrier of one copy of this haplotype had 1.79 times higher breast cancer risk relative to women who did not carry it, much stronger than the best SNP rs47995220 alone (OR = 1.23; 95% CI = 1.11-1.45). These three novel haplotypes found on chromosomes 1, 4 and 18 were further verified with comparison to the imputed SNPs based on the 1000 Genomes Project released data within the same chromosomal regions. None of the aforementioned novel haplotype-specific associations could have been revealed by imputed SNPs (Figure 3A–C). As shown in the Manhattan plots contrasting the haplotype effects with that of the imputed SNPs, the most significant haplotypes were independent of the neighboring clusters of imputed SNPs; no adjacent SNPs achieved comparable significance as the top haplotypes did. These novel haplotypes were not confounded by local ancestry inferred from neighboring SNPs either (Table S5). The test statistics stayed largely unchanged after further adjusting for the local ancestry in addition to the global ancestry for a finer correction for population admixture. Among the remainder of the top 10 independent regions with haplotype global test p-values less than 1.51×10^−5^, the significance levels of the top individual haplotypes and SNPs were very close for chromosomes 3, 5 and 10, implying that the noticeable haplotype effects shown on the Manhattan plots can be mostly credited to the genotyped SNPs (Figures S3 A–C). On the rest of the chromosomes, the top SNPs were more significant than any inferred haplotypes, so that the haplotypes did not contribute more information towards genetic association tests in those regions than SNPs themselves.

**Table 2:** The most significant individual haplotypes identified in the extended regions on chromosomes 1, 4 and 18.

				Unadjusted for SNP effect			Adjusted for SNP effect		
Chromosome	Constituent SNPs	Haplotype	Frequency	OR	95% CI	Hap P	OR	95% CI	Hap P^a^
1	rs9628987,rs2289731,rs12711517,	AGCTG	0.24	0.81	(0.74–0.89)	5.09E–06	0.82	(0.74–0.91)	1.36E–04^c^
	rs2305016,rs7535752								
	**SNP adjusted** b								
	rs12711517; T, 0.36; 1.11 (1.03–1.20);p = 9.88E–03								
4	rs17435444,rs13116936	AG	0.64	1.23	(1.13–1.33)	3.37E–07	1.74	(1.26–2.39)	7.54E–04^c^
	rs13116936; T, 0.34; 0.84 (0.77–0.91);p = 1.09E–05								
18	rs7233920,rs4799278,rs12605634,	AACGTT	0.03	1.72	(1.32–2.25)	6.96E–05	1.79	(1.36–2.34)	2.42E–05
	rs4799520,rs7238528,rs17702736								
	rs4799520; A, 0.09; 1.23 (1.11–1.45);p = 3.66E–04								

a the p-value of LR test of the haplotype-specific effect after adjustment for the best SNP contained in that haplotype.

b the rs number, risk allele and its frequency, Odds Ratios and 95% CI, and the p-value of the SNP that is adjusted for in the LR test are presented.

c There are no individual haplotypes significant at 1.0E-4 in this region after adjustment for the best contained SNP. Instead the most significant haplotype is reported for the sake of completeness.

**Figure 4:**
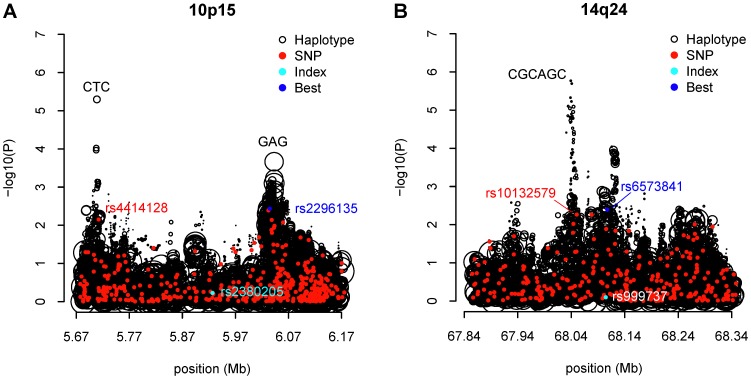
Two known breast cancer risk regions 10p15 and 14q24 exhibit putative haplotype effects. (A) 5.67–6.17 Mb region at 10p15; (B) 67.84–68.34 Mb region at 14q24. Black circles denote individual haplotypes, the sizes of which are proportional to their haplotype frequencies. Red dots denote genotyped SNPs within the same region. Blue dot shows the most significant SNP. Cyan dot denotes the known breast cancer risk SNP identified by previous GWAS.

**Figure 5:**
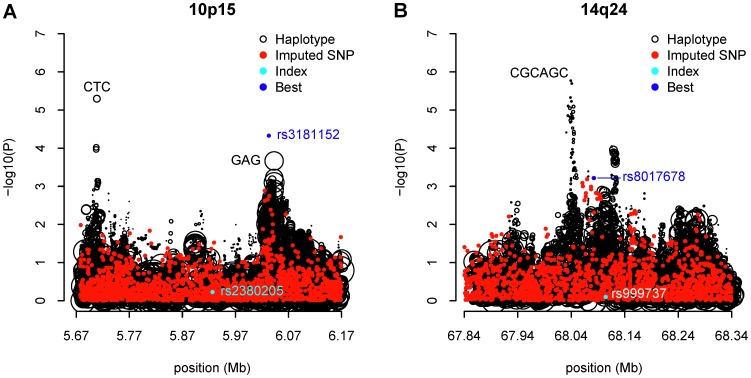
Comparison of the significance of individual haplotypes with imputed SNPs in 10p15 and 14q24. (A) 5.67–6.17 Mb region in 10p15; (B) 67.84–68.34 Mb region in 14q24. Black circles denote individual haplotypes, the sizes of which are proportional to their haplotype frequencies. Red dots denote imputed SNPs within the same region. Blue dot shows the most significant imputed SNP. Cyan dot denotes the known breast cancer risk SNP identified by previous GWAS.

As noted by Chen et al [23], the endeavor to replicate the significance of the known GWAS hits using the AABC data was largely unsuccessful, implying the risk loci for breast cancer found in other GWAS, predominantly of European ancestries, may not be the same as in African Americans. For four of the known GWAS SNPs the associations in our African American breast cancer data had a nominally significant p-value less than 0.05 (Table S4), namely rs13387042 at 2q35 (OR = 0.89; 95% CI = 0.82–0.97; p = 0.00713), rs865686 at 9q31 (OR = 0.92; 95% CI = 0.85–0.99; p = 0.0287), rs2981582 at 10q26 (OR = 1.11; 95% CI = 1.03–1.19; p = 0.0087), and rs2363956 at 19p13 (OR = 0.88; 95% CI = 0.82–0.95; p = 8.1×10^−4^). They are all common variants of modest effects in this study with minor allele frequency between 0.07 and 0.49. Across these 21 regions with known breast cancer risk, 10p15 and 14q24 showed potential haplotype effects with the global test p-value less than 1.0×10^−4^, albeit not genome-wide significant. When scrutinizing all possible inferred individual haplotypes of 2–10 SNPs long in the vicinity of the known markers, a 3-SNP haplotype at 10p15, CTC (Position: 5705780–5712025; frequency = 0.22) constituted by rs17141741, rs2386661 and rs4414128 was three orders of magnitude more significant than the most significant individual SNP contained in the haplotype, rs4414128 (unadjusted haplotype p-value = 5×10^−6^ vs. best SNP p-value = 7.08×10^−3^) (Table 3). This haplotype was associated with a 20% reduced risk per copy for breast cancer relative to the women not carrying it. The haplotype-specific effect was almost unchanged after adjustment for both the best contained SNP (rs4414128) and the index marker (rs2380205) (adjusted haplotype OR = 0.81, 95% CI = 0.72–0.91, p = 2.16×10^−4^). The haplotype signal was two or three orders of magnitude more significant than any of the remaining individual SNPs adjacent to that haplotype, as shown from the leftmost haplotype signal peak in Figure 4A. When further compared to the 1 KGP imputed SNPs in the same region, this CTC haplotype was still independent of the imputed SNPs (Figure 5A). The imputed SNPs residing within close proximity had similar significance levels to that of the genotyped SNPs (Figure 4A vs. Figure 5A), which emphasized that haplotype effect was unlikely to be explained by SNP imputation either. Another 3 SNP haplotype GAG (Position: 6042374–6043841; frequency = 0.60) was stronger than any genotyped SNPs. However, we found an imputed SNP (rs3181152; risk allele: G; frequency: 0.45; p = 4.72×10^−5^) that fell on this haplotype and was an even stronger predictor of risk. The analysis of individual haplotype effects also identified a new region at 14q24 containing the known hit rs999737, where the most significant haplotype was CGCAGC (Position: 68033499–68045127; frequency = 0.05) with the unadjusted haplotype p-value over three orders of magnitude less than that of the best contained SNP, rs10132579 (unadjusted haplotype p = 1.69×10^−6^ vs. best SNP p = 9.55×10^−3^) (Figure 4B). It was also noted that this haplotype effect was stable after additional adjustment for rs10132579 and rs999737 (unadjusted OR = 0.60, 95% CI = 0.48–0.74 and the adjusted OR = 0.60 with 95% CI = 0.47–0.77), suggesting approximately a 40% decreased breast cancer risk per copy was associated with this CGCAGC haplotype among the carriers. Taking local ancestry into account did not change the results for either the CTC haplotype on 10p15 or the CGCAGC haplotype on 14q25 (Table S5). There were numerous other individual haplotypes with unadjusted significance between 10^−6^ and 10^−5^ on 8q24 and 19p13. However, these top haplotype effects were indistinguishable from the top SNPs. Once adjusted for the best SNP contained, these haplotypes became insignificant (p>0.05) (Figures S4 A–D).

**Table 3:** The most significant individual haplotypes in 10p15 and 14q24.

				Unadjusted for SNP effect			Adjusted for SNP effect		
Chromsome	SNPs	Haplotype	Frequency	OR	95% CI	Hap P	OR	95% CI	Hap P^a^
10p15	rs17141741,rs2386661,rs4414128	CTC	0.22	0.79	(0.72–0.88)	5.00E–06	0.81	(0.72–0.91)	2.16E–04
	**Known Risk SNP adjusted** b								
	rs2380205; C, 0.42; 0.98 (0.91–1.06); p = 0.5945								
	**Best SNP adjusted** b								
	rs4414128; T, 0.38; 1.11 (1.03–1.21); p = 0.007084								
14q24	rs765899,rs737387,rs2842347,	CGCAGC	0.05	0.6	(0.48–0.74)	1.69E–06	0.6	(0.47–0.77)	4.27E–05
	rs757369,rs10132579,rs2842346								
	rs999737; T, 0.05; 0.98 (0.82–1.17); 0.7994								
	rs10132579; G, 0.37; 0.89 (0.82–0.97); p = 0.009551								

a the p-value of LR test of the haplotype specific effect after adjustment for both the known breast cancer risk SNP and the best SNP contained in that haplotype.

b the rs number, risk allele and its frequency, Odds Ratios and 95% CI, and the p-value for the SNP adjusted in the LR test are presented. For the regions with known breast cancer risk hits, both the known hit and the locally best SNP were adjusted for in the LR test for the independence of haplotype signals.

## Discussion

We implemented a genome-wide haplotype association analysis searching for breast cancer risk susceptibility loci in African American women. To quickly narrow down to potential risk regions, a 5-SNP sliding window approach was applied throughout 22 autosomes. Among approximately 1 million windows, none achieved the genome-wide significance determined by an approximation to the beta distribution of the minimum p-values through 1,000 permutations (p_G_ = 9.94×10^−8^). Only 10 independent chromosomal regions had the haplotype global test p-value less than 1.5×10^−5^. The haplotype AGCTG at chromosome 1∶8,309,317–8,318,147 showed a moderate haplotype effect that was otherwise not captured by association analyses focusing on SNPs. This region overlaps a solute carrier family 45 member 1 gene (*SLC45A1*, position: 8,306,977–8,326,814) that is predominantly expressed in brain tissues and is also seen frequently deleted in brain tumor cells, suggesting a putative role as a tumor suppressor [53], however, the clear picture of its biological mechanism is far from complete. The 2-SNP haplotype AG on chromosome 4∶122,340,944–122,346,258 had a stronger association with the disease than any SNPs in the same region. About 30 kb upstream of it resides TNIP3 gene (Homo sapiens TNFAIP3 interacting protein 3). Both TNIP and TNFAIP proteins were reported to overexpress in human carcinoma cells and suppress the activation of nuclear factor kappa B (NF-κB) [54]. The haplotype AACGTT at chromosome 18∶35,670,316–35,684,521 was associated with increased risk for breast cancer and the haplotype effect was independent of individual SNP effects, although no known genes are found nearby. Therefore, these regions revealed by haplotype analysis are candidates for fine-mapping to locate the casual variants as a first step towards deciphering the true biological functions. Among the 21 known breast cancer risk regions revealed by previous GWAS, 10p15 and 14q24 seem most likely to harbor unknown risk loci based on the suggestive haplotype associations described above.

In previous work, Chen et al. [23] and Siddiq et al. [55] have shown that the genome-wide significance for the 21 known breast cancer SNPs did not replicate in African Americans. The majority of those SNPs were discovered predominately in European populations, with the exception of rs2046219 at 6q25 found in the Han Chinese population. Chen et al have also shown that many of the index risk variants for breast cancer are significant in multiple populations except for African Americans [56]. We confirmed that the most significant SNPs within each known region are all different from the known breast cancer risk SNPs (Table S4). All evidence underscores the different risk association patterns between African Americans and European populations, and limits the generalizability of the previously established significant GWAS hits as well as presents new challenges in the investigation of breast cancer susceptibility loci specifically for African Americans.

The haplotype association tests were based on haplotype dosage estimates inferred by the E-M algorithm from unphased genotypes for unrelated subjects under the assumption of HWE (the estimation step). We substituted the expected haplotype dosages for the unknown true haplotypes and fit these continuous dosage variables into conventional logistic regression models (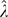
in models 3 through 7) (the substitution step). Even though the haplotype inference from diploid genotypes is not free from uncertainty, the use of these continuous dosages largely correct for the uncertainty derived from haplotype inference and the predictability of haplotypes is quite high, especially when adjacent SNPs are in high LD, a condition that often satisfies in analyses focused on haplotype blocks [38]. This simple expectation-substitution approach [57] has been shown to have a proper control of the type I error rate for the association test when we believe the haplotype dosage estimates have no differential errors between cases and controls [58]. In other words, case-control status is unrelated to the errors in haplotype dosage estimation, which is generally valid when haplotypes are inferred by pooling both cases and controls and the null hypothesis of no significant association between haplotype and disease is true. Several concerns arise when under the alternative hypothesis a few assumptions are no longer true. For instance, if haplotype frequencies in cases and controls are associated with the disease status, failure to account for haplotype uncertainty can lead to estimates biased towards to null [59], [60]. Second, even though the SNPs are in HWE in the general population, it may not be necessarily so in the case-enriched case-control sample so that the estimation of haplotype dosages may not be accurately inferred from the sample’s genotypes. To address these aforementioned issues, Lin et al. [61], [62] proposed a maximum likelihood (ML) method that simultaneously infers haplotype frequencies and regression parameters in the same model. Their method yields less biased estimates and the confidence intervals of the regression coefficients have better coverage of the true value through simulation data for a variety of settings under the alternative hypotheses. We note however that the superiority of the ML method over the expectation-substitution applies only to scenarios where the true magnitude of association is very large, i.e., β = 0.9 (OR = 2.5). Such large effects seem to be rare in GWAS of either common SNPs or common haplotypes studied here. Another simulation analysis [59] also verified that in practical settings where a haplotype block formed by a small number of SNPs with limited haplotype diversity, the bias was minimal and the empirical confidence intervals had appropriate coverage of the true value. More importantly, the performances of the maximum likelihood method and the expectation-substitution were almost indistinguishable, implying the expectation-substitution is robust to reasonable departure from the assumptions. Therefore, substituting the inferred haplotype dosages in the regression model still retains good statistical properties in most practical contexts of haplotype association tests. If haplotypes with greater risk effect were of interest, the simultaneous maximum likelihood method would be preferable.

We may not have had enough statistical power to identify significant rare haplotypes or modest to weak haplotype effects despite our large sample size. Haplotypes of less than 1% frequency were unaddressed in our analyses mainly due to the intrinsic difficulty and unreliability of inference of those rare haplotypes. Uncommonly short or long haplotypes in the genome compromise our 5-SNP sliding windows flexibility to identify them in the haplotype global test. It is possible that constructing a larger window may capture more haplotype variety such that some rare haplotypes can be taken into account. Nonetheless, concerns of computing efficiency arise as the number of SNPs increases. For example, if the total number of heterozygous SNPs in each haplotype block is *m*, there could be 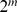 possible haplotypes and thus 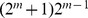 possible haplotype pairs being summed over in the E-M algorithm for each subject. The number grows exponentially, exacerbating the feasibility of implementing the algorithm. Even though in reality, the number of possible haplotypes may just be a fraction of 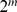, the same idea still applies. Qin et al [63] proposed the partition ligation E-M algorithm by breaking up a sequence of SNPs into smaller pieces, each including 5–10 markers. In our study, in order to maximize the coverage of all genotyped SNPs, a 5-SNP window was adopted to construct haplotype blocks and haplotype global test was employed therein. Arguably, varying window sizes are capable of reflecting varying degrees of LD in the data [64], [65]: more SNPs should be included in the same haplotype block when they are in regions of extensive LD and fewer SNPs should be portioned together given limited LD structure. However, it is difficult to identify regions of high and low LD and alter the window sizes accordingly across the entire genome with high precision. It was recommended by Mathias et al [21] that smaller window sizes be run prior to larger windows. We employed a strategy that both quickly narrows down to potentially important regions through the universal 5-SNP sliding windows and permits the flexibility of detecting underlying haplotypes of 2–10 SNPs long residing in those regions. The choice of 5-SNP window roughly agrees with the overall average haplotype block sizes for people of African ancestry, in which the total number of the haplotype blocks longer than 10 SNPs (∼25 kb) should not be unexpectedly large [9]. Larger windows may improve the ability to identify unknown haplotype effects. However, if a haplotype effect existed in a 10-SNP block, it would have been at least partly captured by at least a few of a series of 5-SNP blocks. Note that this should never be used as a one-size-fits-all solution since the SNP density, underlying haplotype diversity, and populations under investigation can be fundamentally different from study to study. A similar exploration of the choice of the average window size is suggested prior to applying the sliding windows approach in other groups with different LD patterns.

One drawback in the use of overlapping sliding windows is the difficulty of making correct inference of the type I errors. Obviously, overlapping windows were not independent. A naïve application of Bonferroni adjustment would incur overly conservative significance levels and the power to find true positive associations would also be compromised. Permutation tests have been shown to be capable of drawing the significant threshold directly from the experimental data [66] and serve as the gold standard in the comparison of performances of various multiple testing adjustments [67]. Nevertheless, permutation tests are computationally very intensive and time-consuming. One thousand permutations in a genome-wide haplotype analysis can take weeks to months to finish in light of large sample sizes, haplotype inference, and association testing. Numerous innovative recommendations [67]–[71] have been proposed and each has its own merits. One category among those approaches incorporates the computation of the effective number of independent tests: n_eff_ and use of n_eff_ in Bonferroni correction. n_eff_ can be inferred from the beta distribution of the minimum p-values from permutation replicates. We conjectured that in our African American sample the true number of effective tests for chromosome 22 lies somewhere between 7,400 and 8,300, covering half the number of total overlapping windows. So the permutation test implies that approximately 50% of total sliding windows can be considered independent and therefore a modified Bonferroni correction can be used readily.

In summary, we applied a 5-SNP sliding window approach to perform genome-wide haplotype association analysis and identified three novel regions with potential interest for further investigation and validation. Two of 21 known breast cancer risk regions established in previous GWAS, namely 10p15 and 14q24, exhibited moderate haplotype effects and warrant additional replication work to confirm their significance in African American women.

## Supporting Information

Figure S1 **The distributions of 5-SNP sliding window sizes shown in cumulative density.** Each colored line denotes the 5-SNP sliding window sizes on each chromosome, shown as cumulative density of window sizes from the smallest to the biggest. The black curve shows the average cumulative density across 22 autosomes. The 1, 25, 50, 75, 90, and 99 percentile of the average window size are 1, 5, 9, 14, 20 and 32 kb, respectively.
(TIFF)

Figure S2 **Comparison of the significance of haplotype blocks and SNPs on chromosome 5 (97.3** **Mb –97.8** **Mb).** Black dots represent the significance of haplotype blocks by the global test. Red circles denote the significance of SNPs in the same chromosomal region. All top five haplotype blocks overlap each other and contain SNP rs6882564, which was severely out of Hardy-Weinberg Equilibrium.
(TIFF)

Figure S3 **Comparison of the significance of individual haplotypes with the most significant SNPs in three regions on chromosomes 3, 5 and 10.** These three regions, namely, (A) chr3∶7,220,000-7,280,000; (B) chr5∶142,326,000- 142,371,000; and (C) chr10∶115,105,000-115,124,000 had small p-values in the genome-wide haplotype association analysis. Black circles denote individual haplotypes, the sizes of which are proportional to their haplotype frequencies. Red dots denote genotyped SNPs within the same region. Blue dot shows the most significant SNP. The observed top individual haplotype effects were mostly due to the top SNPs.
(TIFF)

Figure S4 **Comparison of the significance of individual haplotypes with genotyped and imputed SNPs in 8p24 and 19p13.** (A),(C) 125.99-129.99 Mb region in 8q24; (B),(D) 17.00–17.50 Mb region in 19p13. Black circles denote individual haplotypes, the sizes of which are proportional to their haplotype frequencies. Red dots denote genotyped SNPs in (A),(B) and imputed SNPs in (C),(D) within the same region of haplotypes. Blue dot shows the most significant genotyped SNP in (A),(B) and the most significant imputed SNP in (C),(D). Cyan dot in (C),(D) denotes the known breast cancer risk SNP identified by previous GWAS.
(TIFF)

Table S1 **Descriptive characteristics of nine studies constituting the African American Breast Cancer study.**
(DOC)

Table S2 **Comparison of the distributions of haplotype block sizes by Gabriel’s method and the 5-SNP sliding window approach.**
(DOC)

Table S3 **Top 10 independent regions defined by 5-SNP sliding windows with the global haplotype test p<1.0E-4.**
(DOC)

Table S4 **The index (Known risk for breast cancer) SNPs and the most significant (best) SNP in 21 known breast cancer risk regions in this study.**
(DOC)

Table S5 **Comparison of the important haplotype associations identified in the analysis with further adjustment for local ancestry.**
(DOC)
