# Supplementary figures and images for: A Genome-Wide Scan for Breast Cancer Risk Haplotypes among African American Women
Source: PLoS One. 2013 Feb 28;8(2):e57298. doi: 10.1371/journal.pone.0057298 (PMC3585353; doi:10.1371/journal.pone.0057298)

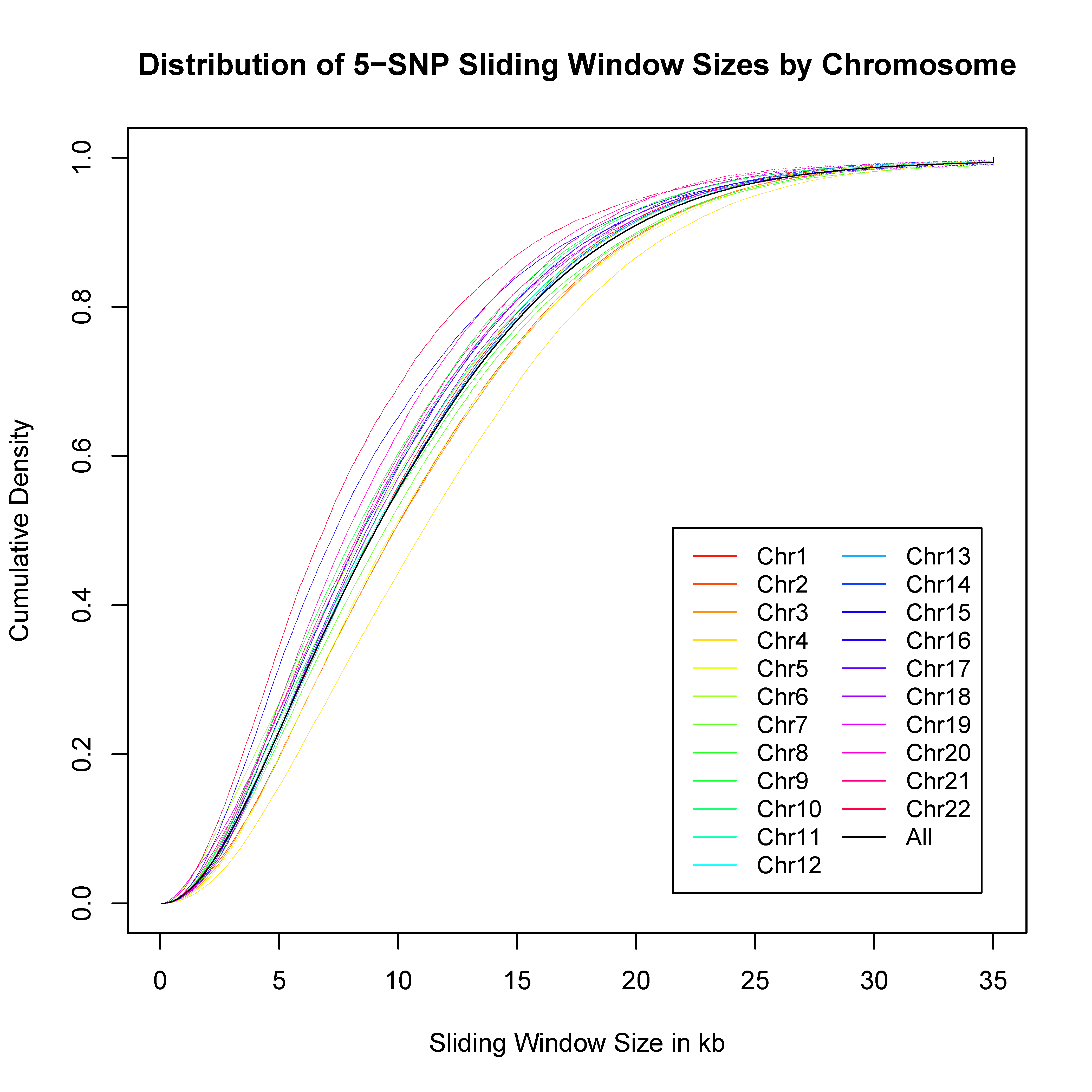

Supplement: Figure S1 — The distributions of 5-SNP sliding window sizes shown in cumulative density. Each colored line denotes the 5-SNP sliding window sizes on each chromosome, shown as cumulative density of window sizes from the smallest to the biggest. The black curve shows the average cumulative density across 22 autosomes. The 1, 25, 50, 75, 90, and 99 percentile of the average window size are 1, 5, 9, 14, 20 and 32 kb, respectively. (TIFF) [file pone.0057298.s001.tiff]

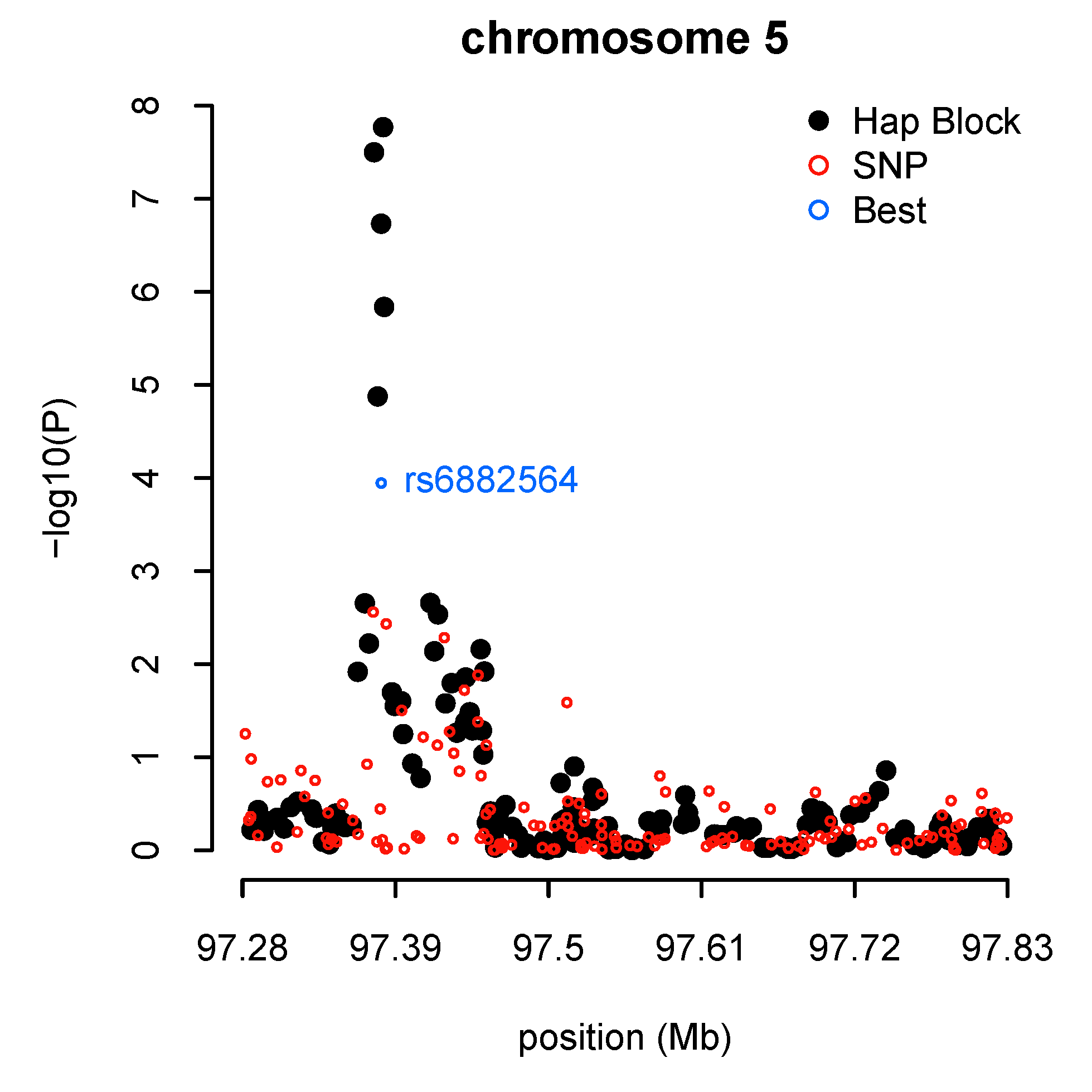

Supplement: Figure S2 — Comparison of the significance of haplotype blocks and SNPs on chromosome 5 (97.3 Mb –97.8 Mb). Black dots represent the significance of haplotype blocks by the global test. Red circles denote the significance of SNPs in the same chromosomal region. All top five haplotype blocks overlap each other and contain SNP rs6882564, which was severely out of Hardy-Weinberg Equilibrium. (TIFF) [file pone.0057298.s002.tiff]

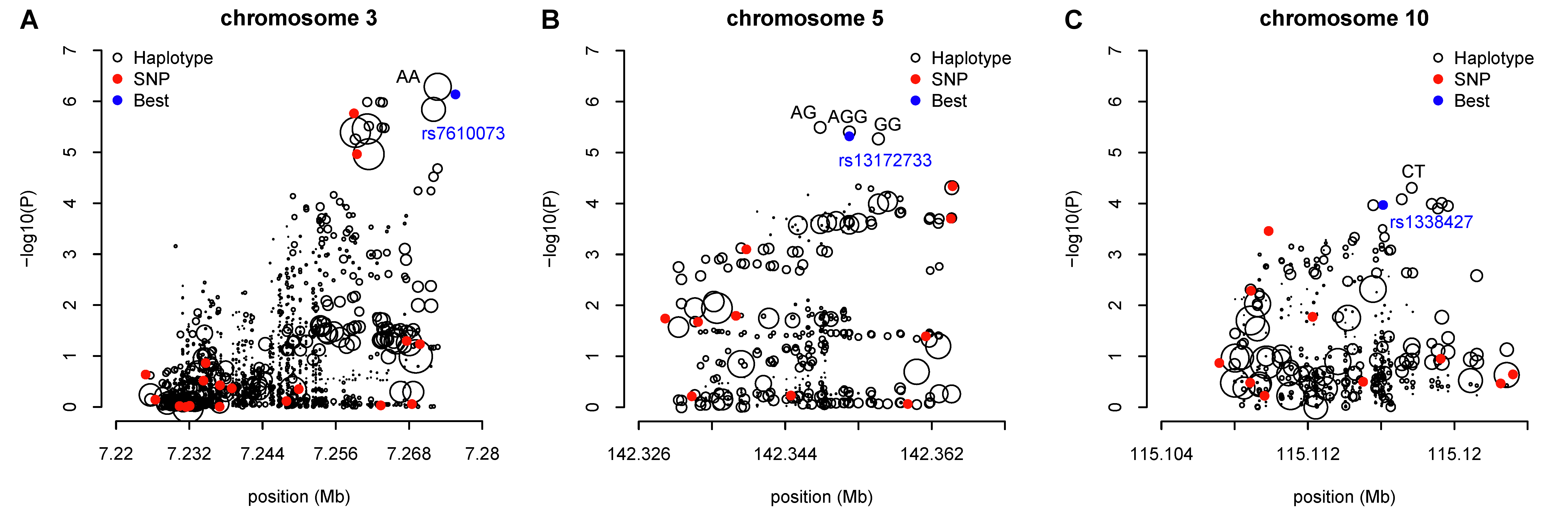

Supplement: Figure S3 — Comparison of the significance of individual haplotypes with the most significant SNPs in three regions on chromosomes 3, 5 and 10. These three regions, namely, (A) chr3∶7,220,000-7,280,000; (B) chr5∶142,326,000- 142,371,000; and (C) chr10∶115,105,000-115,124,000 had small p-values in the genome-wide haplotype association analysis. Black circles denote individual haplotypes, the sizes of which are proportional to their haplotype frequencies. Red dots denote genotyped SNPs within the same region. Blue dot shows the most significant SNP. The observed top individual haplotype effects were mostly due to the top SNPs. (TIFF) [file pone.0057298.s003.tiff]

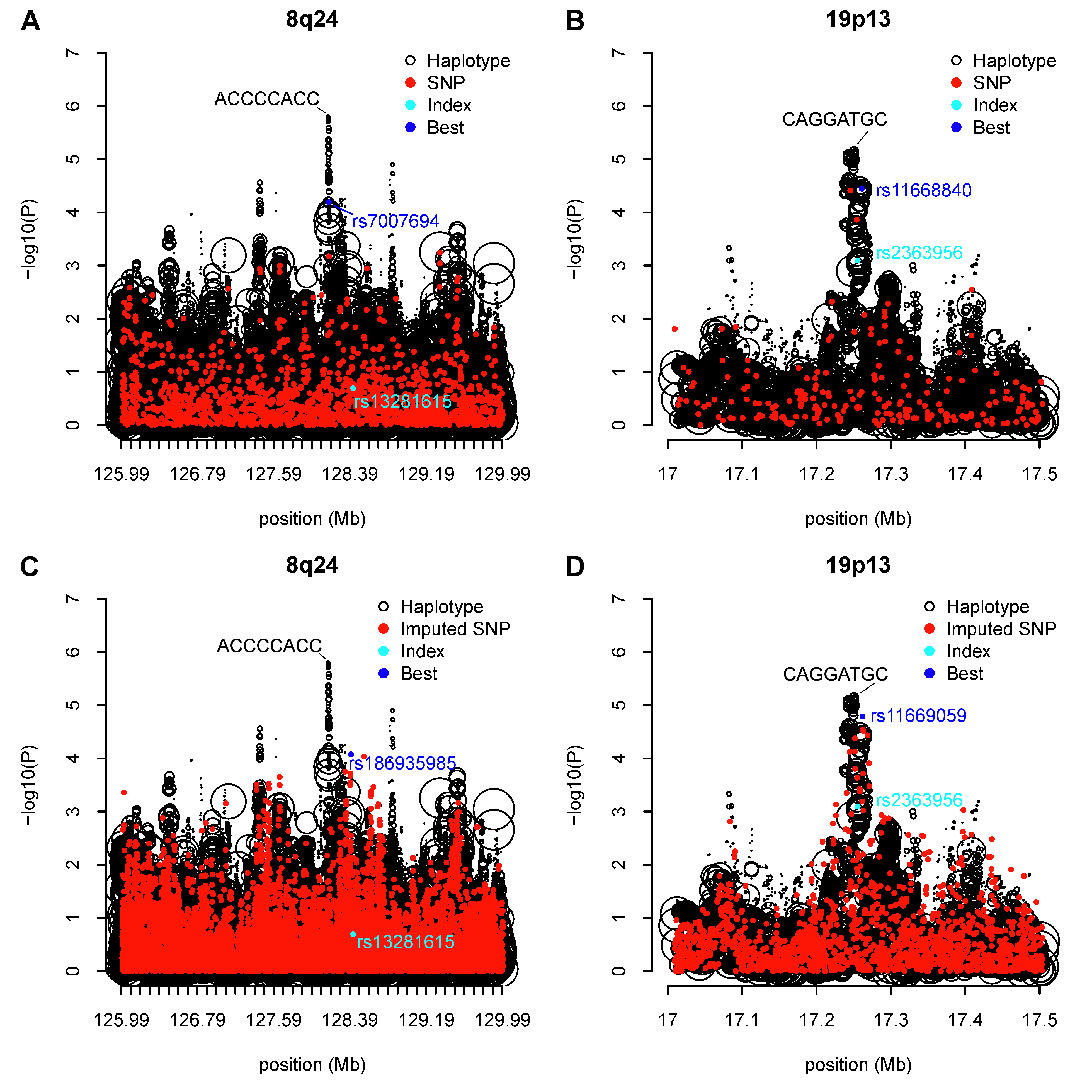

Supplement: Figure S4 — Comparison of the significance of individual haplotypes with genotyped and imputed SNPs in 8p24 and 19p13. (A),(C) 125.99-129.99 Mb region in 8q24; (B),(D) 17.00–17.50 Mb region in 19p13. Black circles denote individual haplotypes, the sizes of which are proportional to their haplotype frequencies. Red dots denote genotyped SNPs in (A),(B) and imputed SNPs in (C),(D) within the same region of haplotypes. Blue dot shows the most significant genotyped SNP in (A),(B) and the most significant imputed SNP in (C),(D). Cyan dot in (C),(D) denotes the known breast cancer risk SNP identified by previous GWAS. (TIFF) [file pone.0057298.s004.tiff]
