# Supplementary material for: A Genome-Wide Scan for Breast Cancer Risk Haplotypes among African American Women
Source: PLoS One. 2013 Feb 28;8(2):e57298. doi: 10.1371/journal.pone.0057298 (PMC3585353; doi:10.1371/journal.pone.0057298)
Supplement: Table S1 — Descriptive characteristics of nine studies constituting the African American Breast Cancer study. (DOC) [file pone.0057298.s005.doc]

**Table S1. Descriptive characteristics of nine studies constituting the African American Breast Cancer study.**

| **Study** |  | | | | | | | | | |
| --- | --- | --- | --- | --- | --- | --- | --- | --- | --- | --- |
|  | MEC**^a^** | CARE | WCHS | SFBCS | NC-BCFR | CBCS**^b^** | PLCO | NBHS | WFBC | Total |
| Cases/Controls | 694/990 | 357/215 | 261/239 | 165/220 | 424/50 | 635/589 | 56/116 | 304/182 | 120/144 | 3,016/2,745 |
| Age**^c^** | 67/68 | 49/48 | 51/51 | 54/54 | 51/50 | 50/50 | 68/67 | 54/52 | 54/55 | 55/58 |
| **First degree family history of breast cancer in Cases, %/Controls, %^d^** | | | | | | | | | | |
| Yes | 21/13 | 11/8 | 16/8 | 41164 | 31/12 | 15/11 | 14/8 | 19/10 | 17/10 | 17/11 |
| No | 74/81 | 84/90 | 84/92 | 91/88 | 69/88 | 82/85 | 84/89 | 80/90 | 83/90 | 80/87 |
| **Estrogen receptor (ER), N (%)^d^** | | | | | | | | | | |
| Positive | 408(59) | 183(51) | 131(51) | 84(51) | 219(52) | 272(43) | 14(25) | 143(47) | 66(55) | 1520(50) |
| Negative | 176(25) | 130(36) | 80(31) | 50(30) | 121(29) | 317(50) | 6(11) | 65(21) | 43(36) | 988(33) |
| **Progesterone receptor (ER), N (%)^d^** | | | | | | | | | | |
| Positive | 292(42) | 144(40) | 105(40) | 76(46) | 196(46) | 252(40) | 13(23) | 113(37) | 48(40) | 1239(41) |
| Negative | 221(32) | 120(34) | 107(41) | 58(35) | 141(33) | 335(53) | 7(13) | 94(31) | 61(51) | 1144(38) |

a MEC includes 556 African American cases (544 invasive and 12 in situ) and 1,003 African American controls. Additionally, 178 African American breast cancer cases diagnosed in Los Angeles Country but outside of the original MEC cohort were augmented to the study.

b Cases of CBCS were identified by rapid case ascertainment system with the North Carolina Central Cancer; controls were gathered from the North Carolina Division of Motor Vehicle and United States Health Care Financing Administration beneficiary lists.

c Ages are expressed in median.

d Not all percentages add up to 100% due to missing ER status for some individuals
