# Supplementary material for: A Genome-Wide Scan for Breast Cancer Risk Haplotypes among African American Women
Source: PLoS One. 2013 Feb 28;8(2):e57298. doi: 10.1371/journal.pone.0057298 (PMC3585353; doi:10.1371/journal.pone.0057298)
Supplement: Table S2 — Comparison of the distributions of haplotype block sizes by Gabriel’s method and the 5-SNP sliding window approach. (DOC) [file pone.0057298.s006.doc]

**Table S2. Comparison of the distributions of haplotype block sizes by Gabriel’s method and the 5-SNP sliding window approach.**

| **Size (kb)** | **Gabriel’s^a^ (%)** | **5-SNP sliding window (%)** |
| --- | --- | --- |
| <1 | --**^b^** | 1.18 |
| 1-10 | 57.2 | 54.25 |
| 10-20 | 20.0 | 35.53 |
| 20-50 | 17.2 | 8.80 |
| >50 | 5.5 | 0.26 |

a The results were obtained by implementation of Gabriel’s method of haplotype block definition in Haploview [36].

b Haplotype blocks with sizes shorter than 1kb were not considered by Wang et al. [35]
