# Supplementary material for: A Genome-Wide Scan for Breast Cancer Risk Haplotypes among African American Women
Source: PLoS One. 2013 Feb 28;8(2):e57298. doi: 10.1371/journal.pone.0057298 (PMC3585353; doi:10.1371/journal.pone.0057298)
Supplement: Table S3 — Top 10 independent regions defined by 5-SNP sliding windows with the global haplotype test p<1.0E-4. (DOC) [file pone.0057298.s007.doc]

**Table S3. Top 10 independent regions defined by 5-SNP sliding windows with the global haplotype test p < 1.0E-4.**

| **Chr** | **Starting Position** | **Ending Position** | **Starting SNP** | **Ending SNP** | **P** | **Independent Region** | | **Extend Region** | | **No. of SNPs in Ext. Reg.** | **No. of Imputed SNPs in Ext. Reg.** | **Common^b^/All haplotypes in Ext. Reg.** | **Cumulative frequencies of common haplotypes** |
| --- | --- | --- | --- | --- | --- | --- | --- | --- | --- | --- | --- | --- | --- |
| 1 | 8309317 | 8318147 | rs9628987 | rs7535752 | 4.53E-06 | 8309317 | 8318147 | 8304902 | 8322562 | 8 | 103 | 21/81 | 0.909 |
| 3^a^ | 7237014 | 7258969 | rs6797852 | rs1810320 | 3.49E-06 | 7237014 | 7263365 | 7223839 | 7276541 | 19 | 233 | 19/1109 | 0.352 |
| 3^a^ | 7247914 | 7263365 | rs1472912 | rs7633040 | 1.03E-05 |  |  |  |  |  |  |  |  |
| 4^a^ | 122325743 | 122346258 | rs17051310 | rs13116936 | 2.41E-06 | 122325743 | 122363114 | 122307058 | 122381800 | 18 | 316 | 25/586 | 0.614 |
| 4^a^ | 122330425 | 122356213 | rs921551 | rs4455437 | 2.33E-06 |  |  |  |  |  |  |  |  |
| 4^a^ | 122340944 | 122363114 | rs17435444 | rs6835704 | 1.60E-06 |  |  |  |  |  |  |  |  |
| 5 | 5373256 | 5377983 | rs3806872 | rs16875333 | 3.48E-06 | 5373256 | 5377983 | 5370893 | 5380347 | 8 | 42 | 20/70 | 0.934 |
| 5 | 142337942 | 142359054 | rs37195 | rs13161500 | 1.51E-05 | 142337942 | 142359054 | 142327386 | 142369610 | 11 | 173 | 16/84 | 0.905 |
| 10 | 115109854 | 115119251 | rs7093035 | rs531790 | 1.45E-05 | 115109854 | 115119251 | 115105156 | 115123950 | 11 | 61 | 16/168 | 0.832 |
| 12 | 93056896 | 93067637 | rs11613298 | rs2230754 | 5.19E-06 | 93056896 | 93067637 | 93051526 | 93073008 | 14 | 71 | 29/387 | 0.711 |
| 15 | 84946917 | 84953515 | rs12909713 | rs17626189 | 1.15E-05 | 84946917 | 84953515 | 84943618 | 84956814 | 8 | 76 | 10/50 | 0.964 |
| 17 | 56746161 | 56751231 | rs3785856 | rs10853029 | 7.66E-06 | 56746161 | 56751231 | 56743626 | 56753766 | 7 | 39 | 6/43 | 0.974 |
| 18 | 35670316 | 35683522 | rs7233920 | rs7238528 | 1.09E-05 | 35670316 | 35683522 | 35663713 | 35690125 | 8 | 44 | 9/39 | 0.986 |

a This sliding window overlaps with other ones on the respective chromosome.

b Common haplotypes refer to the ones > 1% frequent in the sample; while all haplotypes refer to all possible inferred haplotypes compatible with the observed genotypes.
