# Supplementary material for: A Genome-Wide Scan for Breast Cancer Risk Haplotypes among African American Women
Source: PLoS One. 2013 Feb 28;8(2):e57298. doi: 10.1371/journal.pone.0057298 (PMC3585353; doi:10.1371/journal.pone.0057298)
Supplement: Table S4 — The index (Known risk for breast cancer) SNPs and the most significant (best) SNP in 21 known breast cancer risk regions in this study. (DOC) [file pone.0057298.s008.doc]

Table S4. The index (Known risk for breast cancer) SNPs and the most significant (best) SNP in 21 known breast cancer risk regions in this study.

| **Chromosome** | **Region** | **Region** |  | **Index SNP** |  | **Minor** | **Major** |  |  |  |  |  |
| --- | --- | --- | --- | --- | --- | --- | --- | --- | --- | --- | --- | --- |
| **band** | **Start** | **End** | **Size kb^a^** | **Best SNP** | **Position** | **Allele** | **Allele** | **MAF** | **HWE** | **OR** | **95% CI** | **P** |
| 1p11 | 120732136 | 121232136 | 500 | rs11249433 | 120982136 | G | A | 0.13 | 0.44 | 1.01 | 0.90-1.14 | 0.84 |
|  |  |  |  | rs2090841 | 120948602 | A | C | 0.21 | 0.95 | 0.93 | 0.84-1.02 | 0.124 |
| 2q35 | 217364077 | 217864077 | 500 | rs13387042 | 217614077 | G | A | 0.27 | 0.89 | 0.89 | 0.82-0.97 | 0.00713 |
|  |  |  |  | rs2372943 | 217612021 | A | G | 0.14 | 0.61 | 0.84 | 0.75-0.93 | 0.00133 |
| 3p24 | 27013344 | 27641017 | 628 | rs4973768 | 27391017 | T | C | 0.4 | 0.06 | 1.04 | 0.96-1.13 | 0.312 |
|  |  |  |  | rs12636719 | 27089601 | C | A | 0.09 | 0.91 | 0.84 | 0.73-0.96 | 0.0102 |
| 5p12 | 44432110 | 45432110 | 1000 | rs4415084 | 44698272 | C | T | 0.37 | 0.16 | 0.98 | 0.90-1.06 | 0.547 |
|  |  |  |  | rs17343612 | 45322115 | C | T | 0.01 | 0.45 | 0.59 | 0.41-0.85 | 0.00493 |
| 5q11 | 55817641 | 56317641 | 500 | rs889312 | 56067641 | C | A | 0.34 | 0.02 | 1.07 | 0.99-1.16 | 0.084 |
|  |  |  |  | rs16886113 | 56030792 | G | T | 0.11 | 0.68 | 1.28 | 1.13-1.44 | 6.49E-05 |
| 6q14 | 81999828 | 82499828 | 500 | rs17530068 | 82249828 | C | T | 0.07 | 0.37 | 1.04 | 0.89-1.21 | 0.63 |
|  |  |  |  | rs12664410 | 82428442 | A | G | 0.04 | 0.81 | 0.79 | 0.65-0.97 | 0.02 |
| 6q25 | 151740059 | 152240059 | 500 | rs2046210^c^ | 151990059 | G | A | 0.4 | c | 1.01 | 0.93-1.09 | 0.88 |
|  |  |  |  | rs10214867 | 152051347 | A | G | 0.02 | 0.63 | 1.49 | 1.15-1.94 | 0.0027 |
| 8q24 | 126000000 | 130000000 | 4000^b^ | rs13281615 | 128424800 | G | A | 0.44 | 0.31 | 1.05 | 0.97-1.13 | 0.204 |
|  |  |  |  | rs7007694 | 128168348 | C | T | 0.25 | 0.77 | 0.84 | 0.77- 0.91 | 6.45E-05 |
| 9p21 | 21802134 | 22302134 | 500 | rs1011970 | 22052134 | T | G | 0.33 | 0.37 | 1.05 | 0.97-1.14 | 0.242 |
|  |  |  |  | rs7049105 | 22018801 | A | G | 0.29 | 0.46 | 1.13 | 1.04-1.23 | 0.00342 |
| 9q31 | 109678299 | 110178299 | 500 | rs865686 | 109928299 | G | T | 0.48 | 0.97 | 0.92 | 0.85-0.99 | 0.0287 |
|  |  |  |  | rs10816584 | 109700413 | G | C | 0.32 | 0.12 | 0.88 | 0.82-0.96 | 0.00257 |
| 10p15 | 5676740 | 6176740 | 500 | rs2380205 | 5926740 | C | T | 0.42 | 0.91 | 0.98 | 0.91-1.06 | 0.595 |
|  |  |  |  | rs2296135 | 6034700 | A | C | 0.26 | 0.92 | 1.14 | 1.04-1.24 | 0.00368 |
| 10q21 | 63698688 | 64198688 | 500 | rs10995190 | 63948688 | A | G | 0.17 | 0.12 | 1.03 | 0.93-1.14 | 0.567 |
|  |  |  |  | rs16917597 | 64093003 | A | G | 0.11 | 0.25 | 0.88 | 0.78-0.99 | 0.0402 |
| 10q22 | 80261154 | 80761154 | 500 | rs704010 | 80511154 | T | C | 0.11 | 0.62 | 0.98 | 0.87-1.12 | 0.816 |
|  |  |  |  | rs12355688 | 80725632 | T | C | 0.22 | 0.72 | 1.24 | 1.13-1.36 | 6.21E-06 |
| 10q26 | 123092307 | 123592307 | 500 | rs2981582 | 123342307 | A | G | 0.47 | 0.01 | 1.11 | 1.03-1.19 | 0.0087 |
|  |  |  |  | rs2981579 | 123327325 | G | A | 0.4 | 0.67 | 0.87 | 0.80-0.94 | 2.77E-04 |
| 11p15 | 1615582 | 2150173 | 535 | rs3817198 | 1865582 | C | T | 0.17 | 0.79 | 0.97 | 0.88-1.08 | 0.619 |
|  |  |  |  | rs3741205 | 2126460 | C | A | 0.32 | 0.93 | 1.11 | 1.02-1.20 | 0.0108 |
| 11q13 | 68787945 | 69287945 | 500 | rs614367 | 69037945 | T | C | 0.13 | 0.93 | 0.96 | 0.86-1.07 | 0.445 |
|  |  |  |  | rs597587 | 69112459 | A | G | 0.48 | 0.18 | 0.86 | 0.80-0.93 | 2.35E-04 |
| 14q24 | 67854435 | 68354435 | 500 | rs999737 | 68104435 | T | C | 0.05 | 0.07 | 0.98 | 0.82-1.17 | 0.799 |
|  |  |  |  | rs6573841 | 68107274 | T | C | 0.37 | 0.93 | 1.12 | 1.04-1.21 | 0.00404 |
| 16q12 | 50893842 | 51393842 | 500 | rs3803662 | 51143842 | G | A | 0.49 | 0.82 | 1.01 | 0.93-1.09 | 0.832 |
|  |  |  |  | rs3112572 | 51157948 | A | G | 0.22 | 0.95 | 1.19 | 1.08-1.30 | 3.32E-04 |
| 17q22 | 50161470 | 50661470 | 500 | rs6504950^c^ | 50411470 | G | A | 0.34 | c | 1.05 | 0.97-1.14 | 0.19 |
|  |  |  |  | rs4794540 | 50300103 | T | C | 0.18 | 0.85 | 0.88 | 0.80-0.97 | 0.0112 |
| 19p13 | 17000704 | 17505124 | 504 | rs2363956 | 17255124 | G | T | 0.49 | 0.11 | 0.88 | 0.82-0.95 | 8.08E-04 |
|  |  |  |  | rs11668840 | 17260625 | C | T | 0.41 | 1.00E-05 | 0.86 | 0.79-0.92 | 3.61E-05 |
| 20q11 | 31801756 | 32301756 | 500 | rs2284378 | 32051756 | T | C | 0.16 | 0.61 | 1.06 | 0.95-1.17 | 0.3 |
|  |  |  |  | rs6142027 | 31863574 | G | T | 0.06 | 0.86 | 1.2 | 1.03-1.40 | 0.02 |

a Known risk regions are defined as index SNP ± 250kb, or slightly extended to the end of block in which the index SNP is nested.

b 8q24 region was expended to the index SNP ± 2Mb for its abundance of cancer related variants.

c Imputed SNP
