# Supplementary material for: A Genome-Wide Scan for Breast Cancer Risk Haplotypes among African American Women
Source: PLoS One. 2013 Feb 28;8(2):e57298. doi: 10.1371/journal.pone.0057298 (PMC3585353; doi:10.1371/journal.pone.0057298)
Supplement: Table S5 — Comparison of the important haplotype associations identified in the analysis with further adjustment for local ancestry. (DOC) [file pone.0057298.s009.doc]

Table S5. Comparison of the important haplotype associations identified in the analysis with further adjustment for local ancestry.

|  |  |  |  |  | **Unadjusted for SNP effect** | | | **Adjusted for SNP effect** | | |
| --- | --- | --- | --- | --- | --- | --- | --- | --- | --- | --- |
| **Chromosome** | **Constituent SNPs** | **Haplotype** | **Frequency** |  | **OR** | **95% CI** | **Hap P** | **OR** | **95% CI** | **Hap P^a^** |
| 1 | rs9628987,rs2289731,rs12711517, | AGCTG | 0.24 | **Global Ancestry** | 0.81 | (0.74-0.89) | 5.09E-06 | 0.82 | (0.74-0.91) | 1.36E-04 |
|  | rs2305016,rs7535752 |  |  |  |  |  |  |  |  |  |
|  |  |  |  | **Global + Local Ancestry** | 0.81 | (0.74-0.88) | 4.72E-06 | 0.82 | (0.74-0.91) | 1.45E-04 |
|  | **SNP adjusted^b^** | | | | | | | | | |
|  | rs12711517; T, 0.36; 1.11 (1.03-1.20); p=9.88E-03 | | | | | | | | | |
| 4 | rs17435444,rs13116936 | AG | 0.64 | **Global Ancestry** | 1.23 | (1.13-1.33) | 3.37E-07 | 1.74 | (1.26-2.39) | 7.54E-04 |
|  |  |  |  | **Global + Local Ancestry** | 1.23 | (1.14-1.33) | 3.02E-07 | 1.71 | (1.23-2.37) | 1.32E-03 |
|  | rs13116936; T, 0.34; 0.84 (0.77-0.91); p=1.09E-05 | | | | | | | | |  |
| 18 | rs7233920,rs4799278,rs12605634, | AACGTT | 0.03 | **Global Ancestry** | 1.72 | (1.32-2.25) | 6.96E-05 | 1.79 | (1.36-2.34) | 2.42E-05 |
|  | rs4799520,rs7238528,rs17702736 |  |  |  |  |  |  |  |  |  |
|  |  |  |  | **Global + Local Ancestry** | 1.69 | (1.29-2.21) | 1.27E-04 | 1.76 | (1.34-2.30) | 4.12E-05 |
|  | rs4799520; A, 0.09; 1.23 (1.11-1.45); p=3.66E-04 | | | | | | | | | |
| 10p15 | rs17141741,rs2386661,rs4414128 | CTC | 0.22 | **Global Ancestry** | 0.79 | (0.72-0.88) | 5.00E-06 | 0.81 | (0.72-0.91) | 2.16E-04 |
|  |  |  |  | **Global + Local Ancestry** | 0.80 | (0.73-0.89) | 1.64E-05 | 0.81 | (0.72-0.91) | 1.62E-04 |
|  | **Known Risk SNP adjusted^b^** | | | | | | | | | |
|  | rs2380205; C, 0.42; 0.98 (0.91-1.06); p=0.5945 | | | | | | | | | |
|  | **Best SNP adjusted^b^** | | | | | | | | | |
|  | rs4414128; T, 0.38; 1.11 (1.03-1.21); p=0.007084 | | | | | | | | | |
| 14q24 | rs765899,rs737387,rs2842347, | CGCAGC | 0.05 | **Global Ancestry** | 0.60 | (0.48-0.74) | 1.69E-06 | 0.60 | (0.47-0.77) | 4.27E-05 |
|  | rs757369,rs10132579,rs2842346 |  |  |  |  |  |  |  |  |  |
|  |  |  |  | **Global + Local Ancestry** | 0.59 | (0.48-0.74) | 1.51E-06 | 0.60 | (0.47-0.77) | 3.95E-05 |
|  | rs999737; T, 0.05; 0.98 (0.82-1.17); 0.7994 | | | | | | | | | |
|  | rs10132579; G, 0.37; 0.89 (0.82-0.97); p=0.009551 | | | | | | | | | |

a the p-value of LR test of the haplotype specific effect after adjustment for both the known breast cancer risk SNP and the best SNP contained in that haplotype

b the rs number, risk allele and its frequency, Odds Ratios and 95% CI, and the p-value for the SNP(s) adjusted in the LR test are presented. For the novel regions identified by 5-SNP sliding windows, the locally most significant SNP was adjusted for; for the regions with known breast cancer risk hits, both the known hit and the locally best SNP were adjusted for in the LR test for the independence of haplotype signals.
